# Vaccines for microbial eye diseases in the era of data science: opportunities and challenges

**DOI:** 10.3389/fimmu.2026.1805560

**Published:** 2026-09-14

**Authors:** Luyang Jiang, Qibo Du, Ying Zhong, Jinjin He, Youfa Fang, Yumei Yang

**Affiliations:** 1 Eyes Center, Shangyu People’s Hospital of Shaoxing, Shaoxing University, Shaoxing, China; 2 School of Medicine, Shaoxing University, Shaoxing, Zhejiang, China

**Keywords:** artificial intelligence, big data, eye infections, ocular immunology, vaccines

## Abstract

**Background:**

Ocular infectious diseases remain an important cause of preventable visual impairment worldwide, yet vaccine development for eye-specific pathogens has lagged behind systemic infections. This gap reflects the biology of the eye, including immune privilege, mucosal immunity, pathogen diversity, and limited understanding of ocular correlates of protection. Advances in data science, artificial intelligence, genomics, and systems biology are reshaping vaccine research by enabling antigen discovery, immune modeling, and surveillance. However, these approaches have not been integrated into a coherent framework for ocular vaccinology.

**Purpose of the review:**

This review examines how data science and computational methods may help accelerate ocular vaccine development by addressing the biological, immunological, and translational constraints that have limited progress in the field. Rather than providing a descriptive overview of ocular pathogens and vaccine candidates, it proposes a challenge–solution framework linking ocular immunobiology, computational vaccinology, and translational implementation.

**Main themes covered:**

The review is organized around three main themes. First, it outlines the biological and immunological barriers that complicate vaccine development for ocular infections, including immune privilege, mucosal immune constraints, antigenic diversity, and poorly defined correlates of protection. Second, it evaluates how computational approaches, including reverse vaccinology, machine learning-based epitope prediction, structural vaccinology, systems immunology, genomic epidemiology, and real-world data analytics, may support antigen discovery, immune modeling, and population-targeted vaccination strategies. Third, it examines the translational barriers that continue to limit clinical implementation, including inadequate ocular disease models, fragmented ophthalmic datasets, challenges in data standardization and interoperability, regulatory uncertainty, and financial constraints.

**Key conclusions:**

Progress in ocular vaccinology is likely to depend on an integrated, systems-level strategy that combines immunology, ophthalmology, and data science. Computational methods may reduce the cost, time, and uncertainty associated with antigen discovery and candidate prioritization, whereas epidemiological and real-world data may strengthen vaccine targeting, surveillance, and post-implementation evaluation. However, meaningful translation will require ocular-specific immune models, standardized multimodal ophthalmic datasets, improved experimental systems, and iterative validation pipelines that connect *in silico* prediction with biological and clinical evidence. Collectively, these advances could help shift ocular vaccinology from a largely reactive field toward a more predictive and evidence-based approach to preventing vision-threatening infections.

## 1 Introduction

### 1.1 The unmet need for ocular vaccines

Ocular microbial diseases represent a substantial yet insufficiently characterized global health burden, contributing significantly to preventable visual impairment and blindness worldwide. Bacterial, viral, fungal, and parasitic eye infections affect hundreds of millions of people annually, with a disproportionately greater impact in low- and middle-income countries, where poor hygiene, limited access to healthcare, and delayed diagnosis remain common (1). Diseases such as trachoma, onchocerciasis, microbial keratitis, and epidemic viral conjunctivitis not only threaten vision but also impose a substantial socioeconomic burden by reducing productivity, limiting educational opportunities, and diminishing quality of life (2). In high-income settings, ocular infections also place considerable pressure on healthcare systems because of recurrent outbreaks, antimicrobial resistance, and long-term sequelae requiring specialized care (3). Despite their broad clinical and societal impact, microbial eye diseases have received lower priority in the global infectious disease agenda than conditions associated with higher mortality rates (4).

Ocular vaccinology has progressed more slowly than systemic vaccine development, partly because of the eye’s distinct anatomical and immunological features. Although the ocular surface is part of the mucosal immune system, it differs substantially from the gut and respiratory tract (5). To preserve vision, the eye maintains immune privilege, a protective state that limits inflammation that might otherwise damage transparent tissues such as the cornea (6). Both the ocular surface and the internal compartments of the eye are tightly regulated by anti-inflammatory mechanisms that preserve tissue clarity, maintain visual function, limit inflammation, and can reduce the strength of vaccine-induced immune responses (7). As a result, most licensed vaccines provide ocular protection indirectly by preventing systemic infections that may secondarily affect the eye, rather than by directly targeting ocular pathogens (8). Although these vaccines have delivered major public health benefits, important gaps remain in protection against eye-specific infections, contributing to preventable vision loss (9). Advances in immunology, biotechnology, and vaccine delivery platforms now offer new opportunities to revisit these challenges and to consider vaccines as effective tools for preventing ocular infectious diseases (10).

The rapid evolution of data science and artificial intelligence has substantially advanced biomedical research by offering quantitative approaches to address the complexity of ocular infections and host immune responses (11). Technologies such as high-throughput sequencing, machine learning-based epitope prediction, structural modeling, systems immunology, and real-world epidemiological analysis are enabling a shift in vaccine development from largely empirical methods to more rational and predictive strategies (12). The convergence of vaccinology, ophthalmology, and data science provides an interdisciplinary framework for identifying novel antigenic targets, distinguishing protective from pathogenic immune responses, optimizing delivery systems and adjuvant selection, and evaluating vaccine performance at the population level (13). This integrated approach is particularly relevant to ocular diseases, which require both precision and adaptability because of their unique biological features, diverse pathogen spectrum, and uneven global distribution (14).

Existing reviews of ocular vaccinology have largely catalogued pathogens and available interventions without constructing the translational logic that connects the specific immunological barriers of the eye to specific computational solutions, or without critically evaluating why the majority of computationally designed vaccine candidates fail to reach clinical use. The present review fills this gap in three ways: it builds an explicit challenge-solution framework anchored in ocular immunobiology; it provides a cross-pathogen mechanistic comparison that identifies where computational approaches add value and where they remain insufficient; and it critically dissects the translational failure rate of *in silico* vaccine candidates, including the ocular-specific factors that amplify this attrition. This approach distinguishes the review from descriptive surveys and provides analytical depth relevant to researchers, clinicians, and translational scientists (15).

This article is a narrative review. Literature searches were performed in PubMed/MEDLINE, Scopus, and Web of Science, supplemented by Google Scholar for grey literature and preprints. Searches covered publications from January 2000 through May 2026, with no language restriction applied prior to retrieval. Primary search terms included “ocular vaccines,” “eye infection vaccine,” “ocular immunology,” “computational vaccinology,” “reverse vaccinology,” “machine learning epitope prediction,” “systems immunology vaccine,” “immune privilege vaccine,” “mucosal immunity ocular,” “trachoma vaccine,” “adenoviral keratoconjunctivitis vaccine,” “herpetic keratitis vaccine,” “bacterial keratitis vaccine,” “Acanthamoeba vaccine,” “onchocerciasis vaccine,” “digital twin vaccine,” “genomic epidemiology ophthalmology,” and “translational attrition *in silico* vaccine”. Pathogens were selected based on their global burden of ocular morbidity, the existence of at least one published vaccine development effort or computational antigen-discovery study, and representation across bacterial, viral, and eukaryotic pathogen categories. Computational approaches were selected to represent the principal methodological classes relevant to antigen discovery, immune modeling, and population surveillance. Reference lists of key retrieved articles were hand-searched for additional relevant sources. Consistent with the scope and objectives of a narrative review, the selection of studies was guided by scientific relevance, methodological quality, and the need to represent diverse pathogen categories and computational approaches; the aim was critical synthesis and translational analysis rather than exhaustive enumeration.

In this review, ocular vaccines are considered under four categories: direct vaccines targeting primary ocular pathogens (e.g., *Chlamydia trachomatis*, adenovirus, and bacterial keratitis agents), indirectly protective systemic vaccines whose benefits extend to the eye (e.g., measles, rubella, and varicella-zoster), experimental mucosal and local immunization strategies under preclinical investigation, and vaccines with documented ocular adverse events relevant to safety monitoring. This framework organizes the sections that follow, which address the biological and immunological barriers to ocular vaccine development, the limits of conventional approaches, the contributions of data science and computational methods, translational and regulatory constraints, and priorities for future research.

## 2 Biological and immunological barriers in ocular vaccinology

Microbial pathogens are major causes of ocular morbidity and preventable blindness worldwide, affecting populations across diverse geographic and socioeconomic settings. Although the eye is continuously exposed to the external environment, it is protected by multiple defense mechanisms, including the tear film, blink reflexes, and innate and adaptive immune surveillance (16). Pathogens can invade a range of ocular tissues, including the conjunctiva, cornea, uveal tract, retina, and intraocular compartments, causing disorders such as conjunctivitis, keratitis, retinitis, and endophthalmitis, many of which can lead to irreversible visual impairment (17). The biological diversity of ocular pathogens, together with the anatomical and immunological features of the eye, presents substantial challenges for vaccine development (18). **Figure 1** summarizes the major microbial pathogens affecting specific ocular structures and the associated risk factors.

**Figure 1:**
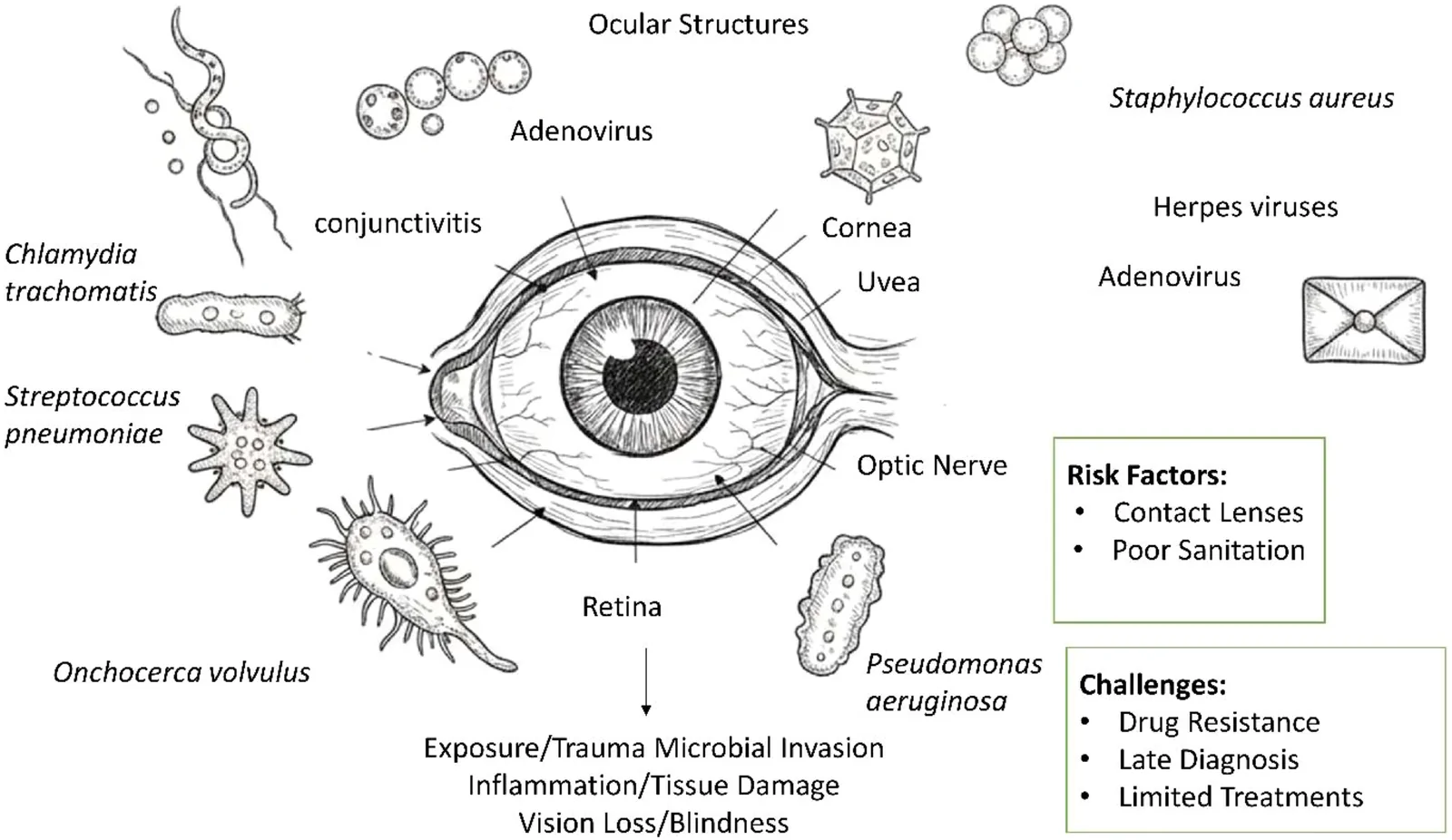
Schematic overview of major ocular structures and representative microbial pathogens associated with ocular infections. The diagram highlights common bacterial, viral, and parasitic agents, routes of exposure, and disease progression leading from inflammation and tissue damage to vision loss, along with key risk factors and clinical challenges.

Ocular pathogens present pathogen-specific and mechanistically diverse barriers to vaccine development. Chlamydia trachomatis, the causative agent of trachoma, poses challenges related to its obligate intracellular biphasic life cycle, strain variability, and the tendency of repeated infection to drive chronic inflammation and scarring rather than durable protective immunity (19). Herpes simplex virus type 1, a major cause of herpetic keratitis, further complicates vaccine design because it establishes lifelong latency in sensory ganglia and undergoes periodic reactivation, while the immune mechanisms required for sustained protection against recurrence remain incompletely defined (20). In adenovirus-associated epidemic keratoconjunctivitis, broad vaccine coverage is hindered by the presence of multiple serotypes and limited cross-protective immunity (21). In bacterial keratitis caused by pathogens such as *Staphylococcus aureus*, *Streptococcus pneumoniae*, and *Pseudomonas aeruginosa*, antigenic heterogeneity, diverse virulence factors, and biofilm-associated persistence complicate antigen selection and cross-strain protection (22). These pathogens produce diverse virulence factors, including exotoxins and proteases, which promote tissue invasion and immune evasion (22). Biofilm formation further enhances persistence and protects bacteria from host defenses and therapeutic interventions (23). Additional complexity is introduced by eukaryotic pathogens such as *Acanthamoeba*, which exists in both active trophozoite and highly resistant cyst forms, and *Onchocerca volvulus*, whose complex life cycle, long-term persistence, and immunomodulatory capacity hinder the identification of protective immune correlates (24). In *Acanthamoeba*, the resistant cyst stage, complex life cycle, and poorly characterized antigenic profile further hinder vaccine development (24). In this context, computational and data-driven approaches may help identify conserved antigens, stage-specific targets, and candidate correlates of protection. **Table 1** summarizes the major pathogen-specific vaccine barriers, relevant computational strategies, and their current validation status.

**Table 1:** Overview of major ocular pathogens highlighting key vaccine challenges, corresponding computational strategies to address these barriers, and the current status of experimental or clinical validation.

Pathogen	Category	Ocular relevance gap	Principal computational contribution	Evidence level	Status
Measles virus	Indirectly protective systemic (licensed)	Systemic IgG does not prevent direct ocular surface infection by unrelated pathogens; protection is conditional on preventing viremia	Real-world effectiveness modeling; geospatial coverage optimization in LMICs	L5	Licensed MMR; proven reduction of measles keratitis
VZV/HZV	Indirectly protective systemic (licensed)	Does not prevent HSV-1 primary ocular infection or recurrent herpetic keratitis; HZO protection incomplete in immunocompromised	Pharmacovigilance AI for ocular adverse event detection; effectiveness modeling	L5	Licensed Shingrix; reduces herpes zoster ophthalmicus
HPV	Indirectly protective systemic (licensed)	Ocular surface neoplasia linkage to HPV types not fully characterized; sIgA not induced at conjunctival surface by systemic route	Epidemiological linkage modeling; conjunctival HPV seroprevalence mapping	L3–L4	Licensed; ocular surface neoplasia endpoint data limited
*C. trachomatis*	Direct ocular vaccine (no licensed product)	Intracellular lifecycle; antigenic variation across serovars; repeated infection drives scarring not immunity	Pan-genome antigen discovery; ML epitope prediction; transcriptomics of protective vs. pathological host response	L1–L2	No licensed vaccine; preclinical candidates only
HSV-1 (keratitis)	Direct ocular vaccine (no licensed product)	Latency in trigeminal ganglion; recurrent keratitis driven by Trm failure not antibody titer; systemic vaccines insufficient	Structural vaccinology (gB/gD epitopes); latency transcriptomics; Trm induction modeling	L2–L3	No licensed ocular vaccine; Phase I systemic data only
Adenovirus (EKC)	Direct ocular vaccine (no licensed product)	Over 30 ocular-tropic serotypes; no cross-neutralizing immunity; no conjunctival sIgA data for any candidate	Computational serotype clustering; deep learning mosaic hexon antigen design; genomic epidemiology	L1–L2	No licensed ocular vaccine; respiratory adenovirus vaccines exist but not ocular
*S. aureus/P. aeruginosa*	Direct ocular vaccine (no licensed product)	Virulence factor diversity; biofilm persistence; antigenic heterogeneity across clinical isolates	Pan-genome comparative genomics; ML-based epitope mapping across strain libraries	L1–L2	*S. pneumoniae* vaccine licensed systemically; ocular-specific candidates exploratory
*Acanthamoeba* spp.	Direct ocular vaccine (no licensed product)	Cyst stage highly resistant; poorly annotated surface proteome; no defined human correlates of protection	Reverse vaccinology; structural modeling of cyst wall proteins	L1	No vaccine; largely exploratory
*Onchocerca volvulus*	Direct ocular vaccine (no licensed product)	Complex multistage lifecycle; long-term immunomodulation; poorly defined ocular protective correlates	Pan-genome analysis; immunoinformatics for conserved larval antigens	L1	Early-stage; no validated ocular candidate

Evidence levels: L1, computational prediction only (*in silico*); L2, *in vitro*/biochemical validation; L3, ocular or relevant animal model data; L4, human immunogenicity data; L5, clinical protection against ocular outcomes.

Despite advances in antimicrobial therapy and population-level control measures, important knowledge gaps continue to hinder the development of vaccines against ocular infections. These include limited understanding of conserved antigenic targets, the immune mechanisms that operate within the eye’s immune-privileged environment, pathogen–host interactions at ocular sites, and the determinants of protective epitopes across genetically diverse strains. In addition, many ocular pathogens remain incompletely characterized with respect to antigenic variation, tissue tropism, and immune evasion strategies (25). Computational approaches, including genome-wide antigen discovery, epitope prediction, structural modeling, and immune simulation, may help address these gaps by identifying conserved vaccine targets across strains and species (26). The integration of bioinformatics, immunoinformatics, and systems biology therefore offers a framework for accelerating the discovery of ocular pathogen-specific vaccine candidates, particularly where conventional vaccine development has been limited (7). These unresolved challenges also help explain why vaccination, although highly effective against many infectious diseases, has been more difficult to translate to ocular protection: the eye presents distinct biological and immunological constraints that differ from those of systemic and respiratory infections.

The conjunctiva and ocular surface constitute a distinct mucosal immune compartment that differs fundamentally from intraocular tissues in its immunological organization and vaccine accessibility. The conjunctival epithelium contains antigen-presenting dendritic cells, intraepithelial lymphocytes, and IgA-secreting plasma cells and is associated with conjunctiva-associated lymphoid tissue (CALT), which functions analogously to other mucosa-associated lymphoid structures (5). The predominant protective immunoglobulin at the ocular surface is secretory IgA (sIgA), produced locally by plasma cells in the conjunctiva and lacrimal gland and transported into the tear film by the polymeric immunoglobulin receptor (27). Systemic intramuscular or subcutaneous vaccination preferentially induces serum IgG but generates only modest sIgA responses at mucosal sites including the conjunctiva, creating an immunological dissociation that limits sterilizing immunity at the primary sites of pathogen entry for conjunctival infections such as adenoviral keratoconjunctivitis and trachoma (27). For pathogens targeting this compartment, intranasal immunization represents the most immunologically rational delivery strategy, as it stimulates the nasopharynx-associated lymphoid tissue and generates conjunctiva-homing IgA-secreting plasma cells via the common mucosal immune system without the tolerability and antigen-retention limitations of direct topical ocular administration (8).

The corneal surface, although contiguous with the conjunctiva, represents a distinct immune environment that requires separate consideration. The normal central cornea is largely devoid of antigen-presenting cells—Langerhans cells and dendritic cells are concentrated in the limbal and peripheral zones—and local protection depends critically on intraepithelial CD8+ tissue-resident memory T cells (Trm), particularly for suppression of herpes simplex virus type 1 reactivation (20, 28). The cornea is avascular, which restricts leukocyte trafficking and limits the local expression of systemically induced immunity. Neither systemic nor conventional mucosal vaccination has yet demonstrated reliable induction of corneal epithelium-homing Trm at concentrations sufficient to prevent recurrent herpetic keratitis, underscoring that this compartment presents vaccine design challenges distinct from both the conjunctival mucosal surface and the intraocular compartments (20).

The anterior chamber constitutes the primary locus of classical ocular immune privilege and presents the most paradoxical challenge for vaccine-induced immunity. Entry of circulating immunoglobulins and complement is severely restricted by the blood-aqueous barrier, formed by the tight junctions of the non-pigmented ciliary epithelium and iris vasculature (29). The aqueous humor itself contains a consortium of constitutively expressed immunosuppressive mediators—including transforming growth factor-beta 2 (TGF-β2), vasoactive intestinal peptide (VIP), alpha-melanocyte-stimulating hormone (α-MSH), and thrombospondin-1—that collectively suppress dendritic cell maturation, T-cell activation, and NK cell function (30). The canonical mechanism of anterior chamber-associated immune deviation (ACAID) extends this privilege to the systemic level: When antigens enter the anterior chamber, resident F4/80+ macrophages transport tolerogenic signals to the spleen, promoting generation of antigen-specific regulatory T cells that suppress Th1 and cytotoxic CD8+ responses systemically (31). The implication for vaccination is paradoxical—systemic immunization with antigens subsequently encountered in the anterior chamber may induce peripheral immune deviation rather than protective recall. Immune privilege in this compartment is therefore not a passive consequence of anatomical isolation but an active immunosuppressive program that is not captured by systemic immune models or current *in silico* prediction frameworks (30, 31).

The retina and vitreous represent the most immunologically restricted compartment of the eye. The blood-retinal barrier—formed by the retinal pigment epithelium and the tight junctions of the retinal vascular endothelium—is among the most selective blood-tissue interfaces in the body, severely limiting entry of circulating immunoglobulins, complement, and lymphocytes into the posterior segment (29). Experimental evidence from herpes simplex retinitis and uveitis models has demonstrated that peripheral serum IgG titers do not reliably predict intraocular protection (32). For pathogens causing intraocular disease—including cytomegalovirus retinitis and bacterial endophthalmitis—systemic vaccination may nonetheless be rational as a strategy to prevent hematogenous seeding and primary viremia before the blood-retinal barrier is breached, even though direct intraocular immune effectors cannot be reliably delivered by systemic or mucosal vaccine routes. Resident regulatory cells including microglia and Müller glia further modulate intraocular immune responses in ways that are incompletely characterized and not currently incorporated into any vaccine design framework.

Together, these biological and pathogen-specific constraints help explain why vaccine development for ocular infections has lagged behind that for systemic pathogens. Understanding these limitations is essential before considering why conventional vaccine strategies have had only limited success and where computational approaches may offer an advantage.

## 3 Limits of conventional vaccine development for ocular infections

Vaccination remains one of the most effective public health strategies for preventing infectious disease, yet its application to ocular infections has been limited and has largely provided protection indirectly rather than through vaccines designed specifically for the eye. This gap reflects the distinct biological, immunological, and translational challenges posed by ocular pathogens and by the immune-regulated environment of ocular tissues, which differ in important ways from those of respiratory and systemic infections. As a result, most currently available vaccines confer ophthalmic benefit primarily by preventing systemic infections that have ocular manifestations, rather than by directly targeting primary ocular pathogens (33). A clear understanding of the current landscape of licensed ocular-relevant vaccines, vaccine candidates under development for major ocular pathogens, and the immunology of the ocular environment is therefore essential for defining the most important gaps and future opportunities in the field (34). **Figure 2** summarizes the principal barriers and current gaps in ocular vaccine development.

**Figure 2:**
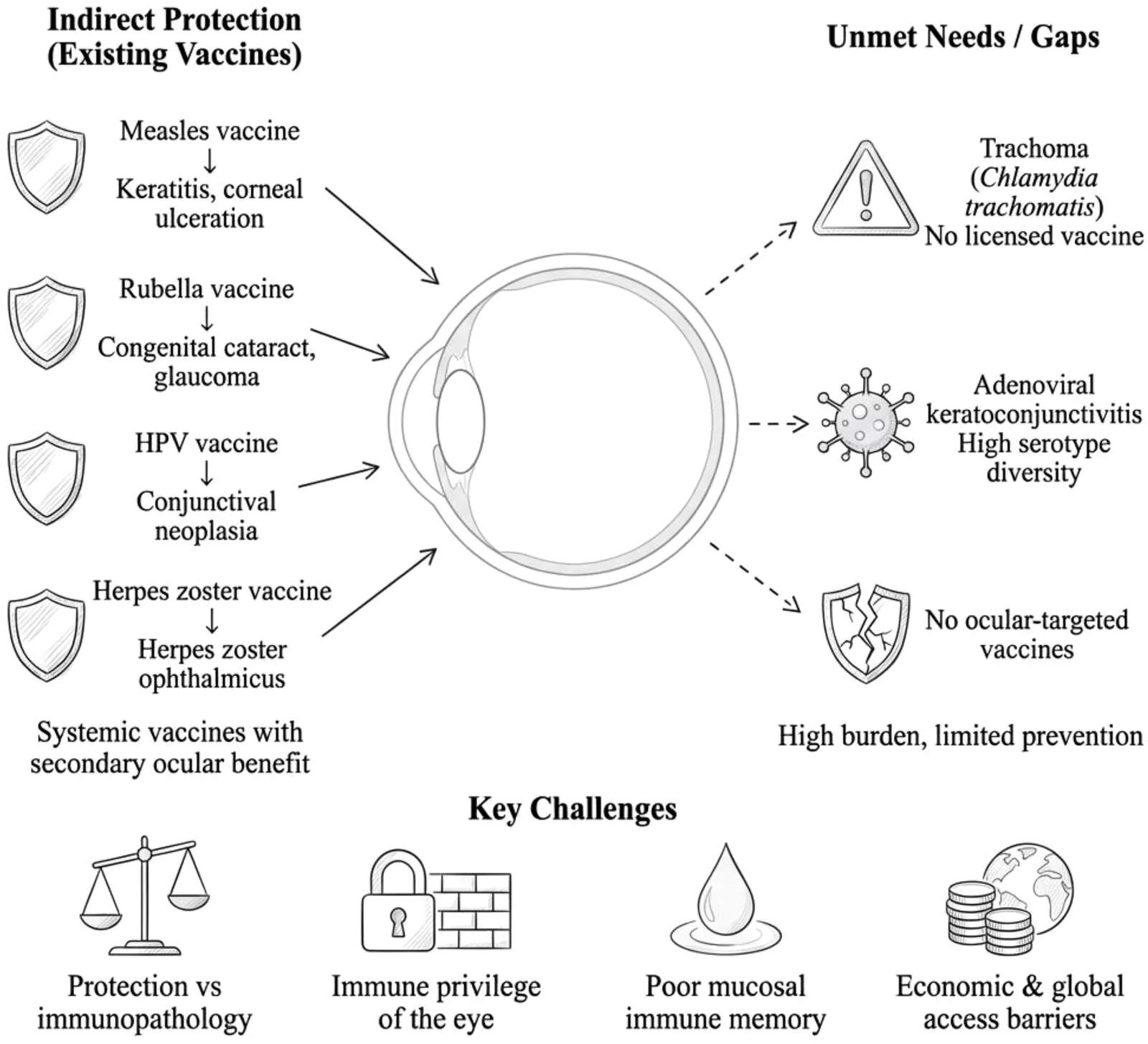
Indirect ocular protection conferred by existing systemic vaccines and the major unmet needs in ocular vaccinology.

### 3.1 Indirectly protective systemic vaccines

Several licensed vaccines confer ophthalmic benefit despite not being designed specifically to prevent ocular infections. Measles vaccination has markedly reduced the incidence of measles-associated keratitis, conjunctivitis, and corneal ulceration, which historically contributed substantially to childhood blindness, particularly in malnourished populations and in settings of vitamin A deficiency (35). However, this protection is indirect and does not extend to unrelated ocular infections such as bacterial keratitis or viral conjunctivitis (36). Similarly, rubella vaccination has nearly eliminated congenital rubella syndrome in many countries, thereby preventing associated cataracts, glaucoma, and retinopathy, but it does not protect against postnatal ocular infections or inflammatory ocular disorders (37). The human papillomavirus vaccine may also provide indirect ophthalmic benefit by reducing the risk of conjunctival papilloma and ocular surface neoplasia linked to oncogenic HPV types, although these manifestations are relatively uncommon (38). However, these are rare ocular complications (39). Herpes zoster vaccination has reduced the incidence of herpes zoster ophthalmicus, but it does not prevent primary infection with herpes simplex virus type 1, a major cause of infectious corneal blindness (40). Collectively, these examples show that systemic vaccination can reduce ocular disease indirectly, but they do not eliminate the need for vaccines directed against primary ocular infections.

### 3.2 Direct ocular vaccine candidates and the rationale for eye-specific immunization strategies

The mechanistic constraints described in Section 2—the blood-ocular barrier limiting IgG entry, the mucosal compartmentalization of sIgA responses, and ACAID-mediated immune deviation—collectively provide a compelling and evidence-based rationale for the development of eye-specific vaccination strategies. Effective protection at the ocular surface requires the local induction of sIgA and the establishment of tissue-resident memory T cells (Trm cells) in the conjunctival and corneal epithelium, both of which are minimally achieved by conventional systemic vaccination routes (28). Experimental evidence from preclinical models of herpetic keratitis has shown that periocular immunization generates superior local protection compared with intramuscular immunization, even when both routes produce equivalent systemic antibody titers (8). Intranasal immunization represents a potentially feasible clinical alternative: by stimulating the nasopharynx-associated lymphoid tissue, intranasal delivery can generate conjunctiva-homing IgA-secreting plasma cells via the common mucosal immune system, inducing ocular mucosal immunity without the safety and practical challenges of direct ocular administration. The design of antigens and delivery platforms that preferentially elicit sIgA and mucosal Trm responses at ocular surfaces, rather than IgG-dominant systemic immunity, constitutes a fundamental and currently underserved priority in ocular vaccinology.

There are currently no licensed vaccines against major ocular infections, including trachoma caused by *Chlamydia trachomatis*, adenoviral keratoconjunctivitis, fungal keratitis, and *Acanthamoeba keratitis* (41). This gap reflects a combination of biological, immunological, and economic barriers to vaccine development. Trachoma remains a major unmet need despite large-scale antibiotic-based control efforts, as reinfection is common and raises concerns regarding long-term effectiveness, antimicrobial resistance, and cost-effectiveness (42). Chlamydial vaccines targeting surface and adhesion proteins have shown partial success in animal models, but translation to safe and effective human vaccines has been challenging, in part because of concerns about immunopathology (42). Ocular adenoviral infections present additional obstacles because of the diversity of circulating serotypes and the absence of vaccines directed against ocular-tropic adenoviruses, despite the availability of vaccines for selected respiratory adenoviruses in restricted populations (43).

Fungal keratitis deserves specific attention as an important and underappreciated unmet need in ocular vaccinology. Caused principally by *Fusarium* species, *Aspergillus* species, and *Candida albicans*—with less common contributions from dematiaceous fungi and emerging resistant species—fungal keratitis is associated with severe corneal scarring, poor visual outcomes, and a high rate of therapeutic failure even with prolonged antifungal therapy (1). The disease disproportionately affects agricultural workers and contact lens wearers in low- and middle-income tropical settings, where delayed diagnosis and limited access to voriconazole or natamycin contribute to blindness. Several biological features complicate vaccine development. The fungal cell wall, composed of beta-glucans, mannans, and chitin, is a potent innate immune activator, but protective adaptive immune responses to corneal fungal infection are incompletely characterized: Th17 responses may contribute to antifungal immunity but can also drive bystander corneal tissue injury, mirroring the immunopathological challenge seen with *C. trachomatis* (44). Licensed antifungal vaccines against systemic candidiasis are in development (e.g., NDV-3A targeting Als3p, completed Phase I trials), and reverse vaccinology has been applied to *Candida* and *Aspergillus* to identify conserved surface antigens; however, none of these candidates has been evaluated in ocular models, and translating systemic antifungal immunity to corneal protection faces the same mucosal compartmentalization barriers described for bacterial pathogens (41). Fungal keratitis therefore represents both an important therapeutic gap and a high-priority target for future ocular vaccine research.

Current limitations in ocular vaccine development can be grouped into several recurring challenges, each of which helps explain why conventional strategies have had limited success and why computational approaches are increasingly being explored. First, antigenic variability and strain diversity remain major barriers. Pathogens such as *Chlamydia trachomatis* and adenoviruses show substantial heterogeneity, whereas organisms such as *Pseudomonas aeruginosa* express diverse virulence determinants that complicate antigen selection. As a result, conventional approaches do not always provide broad or durable protection, whereas reverse vaccinology, pan-genome analysis, and structural epitope mapping may help identify more conserved targets.

Second, generating effective immunity at the ocular surface remains difficult. Rapid tear-mediated antigen clearance and limited local immune induction—arising directly from the mucosal and structural constraints of the ocular surface—reduce the effectiveness of vaccine strategies that perform well in systemic settings. Computational immunology approaches may help guide antigen design toward improved mucosal responses, although their predictive value remains limited by incomplete knowledge of ocular immune mechanisms.

Third, vaccine design must balance protective immunity with the risk of inflammation-mediated tissue damage. Ocular immune privilege preserves visual function by tightly regulating inflammation, but these same mechanisms can constrain vaccine-induced responses. In this context, mathematical and systems-level models may help identify strategies that enhance protection while limiting immunopathology, although experimental validation remains essential.

A fourth challenge lies in vaccine delivery. Systemic immunization does not always generate sufficient local immunity at the ocular surface, whereas direct ocular delivery must overcome tear turnover, epithelial barriers, and tolerability constraints. Computational modeling may support the optimization of delivery platforms such as nanoparticles and mucoadhesive systems, but these predictions still require validation in relevant preclinical models.

A fifth challenge is distinguishing protective immune responses from those associated with disease progression. In trachoma, for example, repeated or dysregulated immune activation may contribute to conjunctival scarring rather than protection. Data-driven approaches may help identify signatures of protection and immunopathology, but progress remains limited by the scarcity of high-quality human ocular immunology data.

Finally, ocular vaccine development continues to face important translational and economic barriers. Infectious causes of blindness are concentrated disproportionately in low- and middle-income countries, where limited commercial incentives may restrict investment in vaccine development (45). Together, these limitations highlight the need for approaches that can prioritize antigens more efficiently, model localized immune responses more realistically, and better integrate pathogen, host, and population-level data. In this context, data science offers the useful framework for advancing ocular vaccine research.

The preferred vaccine delivery route for ocular pathogens differs substantially depending on the anatomical compartment targeted. For conjunctival and corneal surface pathogens, mucosal delivery routes—principally intranasal immunization, which activates the nasopharynx-associated lymphoid tissue and generates conjunctiva-homing IgA-secreting plasma cells via the common mucosal immune system—represent the most immunologically rational strategy for inducing local sIgA and surface Trm (27). Direct topical ocular immunization is limited by rapid tear clearance, low antigen retention, and mucosal tolerogenic mechanisms that favor immune deviation over effector responses (29). Subconjunctival and periocular injection approaches can achieve local antigen deposition but carry risks of local inflammation and tissue injury, particularly in the immune-privileged environment of the anterior chamber. For pathogens causing intraocular disease—including cytomegalovirus retinitis, bacterial endophthalmitis, and toxoplasmic retinochoroiditis—systemic vaccination may be rational as a strategy to prevent hematogenous seeding, although the blood-retinal barrier still limits immunoglobulin access to the vitreous and retina.

## 4 How data science/AI can address specific challenges

### 4.1 Computational tools for antigen discovery and immune modeling

The rapid development of data science and artificial intelligence has reshaped contemporary vaccinology by transforming how antigens are identified, prioritized, and evaluated. As summarized in **Table 2**, these computational approaches offer multiple opportunities to address the biological, immunological, and translational challenges that constrain ocular vaccine development. Traditional vaccine discovery has relied largely on empirical strategies, including attenuation, inactivation, protein purification, and iterative experimental testing; although successful for many infectious diseases, these approaches are often time-consuming, resource-intensive, and less effective for pathogens with marked genetic diversity or complex immune-evasion mechanisms (46). In contrast, bioinformatics and computational biology enable genome-scale antigen discovery, supported by the expanding availability of pathogen whole-genome sequences generated through high-throughput sequencing (47). A central example is reverse vaccinology, first successfully applied to *Neisseria meningitidis*, which identifies candidate antigens directly from genomic data rather than through culture-based and biochemical fractionation approaches (48, 49). Computational pipelines can rapidly annotate open reading frames and predict subcellular localization, secretion signals, and transmembrane domains, thereby prioritizing proteins that are surface exposed or secreted and are therefore more accessible to host immune recognition. Comparative genomics further enables the identification of conserved antigens across strains or serotypes, with the aim of reducing immune escape and broadening potential vaccine coverage (50).

**Table 2:** Opportunities for applying artificial intelligence and data-driven approaches across the ocular vaccine development pipeline.

S. no	Opportunity area	Core method	Ocular application and benefit
1	Antigen discovery	*In silico* genomics & epitope mapping	Designs broad-coverage, multivalent antigens for variable ocular pathogens (e.g., *Adenovirus*, *C. trachomatis*)
2	Immune correlates prediction	Machine learning on multi-omics & clinical data	Defines local ocular protection biomarkers, enabling targeted design and faster clinical trials
3	Delivery system optimization	Computational modeling of nanoparticles/vectors	Enhances ocular surface retention and penetration while minimizing systemic exposure and toxicity
4	Adjuvant selection	AI-guided analysis of immune response data	Selects adjuvants that stimulate protective mucosal immunity without breaking ocular immune privilege
5	Vaccine repurposing	Data mining & network pharmacology analysis	Identifies existing vaccines with potential cross-protective effects, accelerating translational development
6	Integrated development pipeline	Combined computational pipeline (antigen to adjuvant)	Shifts from empirical to rational design, de-risking development for unique ocular challenges
7	Cross-strain conservation analysis	Comparative genomics & evolutionary modeling	Identifies conserved epitopes across pathogen strains for universal vaccine targets
8	Toxicity and safety prediction	*In silico* toxicology & immunogenicity screening	Reduces risk of adverse ocular reactions during early-stage vaccine development
9	Formulation optimization	Computational design of mucosal delivery systems	Creates eye-specific formulations that overcome tear clearance and epithelial barriers
10	Population coverage prediction	HLA-binding prediction algorithms	Ensures vaccine candidates are effective across diverse genetic populations
11	Immune response simulation	Systems immunology & computational modeling	Predicts complex immune interactions specific to ocular tissues before animal testing
12	Clinical trial design support	AI analysis of historical trial data	Optimizes trial endpoints and patient stratification for ocular vaccine studies
13	Disease progression modeling	Machine learning on longitudinal patient data	Identifies optimal vaccination timing and duration of protection for ocular infections
14	Manufacturing process optimization	AI-driven process analytical technology	Improves yield and consistency of complex ocular vaccine formulations

Machine learning has become a major component of modern bioinformatics and now underpins many predictive tasks in vaccine design, extending beyond traditional rule-based approaches (51). One of its most important applications is epitope prediction, a key step in identifying short peptide sequences capable of eliciting B-cell or T-cell responses (52). Earlier epitope-prediction methods relied largely on motif-based rules and physicochemical heuristics, whereas current machine-learning approaches use curated datasets of experimentally validated epitopes to capture more complex patterns of immune recognition. In particular, prediction of major histocompatibility complex binding, a central determinant of T-cell immunity, has been substantially improved by supervised learning methods, including support vector machines, random forests, and deep neural networks (53).

To situate the computational approaches within their actual evidence base, we apply a five-level grading framework: level 1, computational prediction only; level 2, *in vitro* or biochemical validation; level 3, evidence from ocular or relevant animal models; level 4, human immunogenicity data; and level 5, clinical protection against ocular outcomes (**Table 3**). This framework distinguishes approaches that have been experimentally validated in an ocular context from those that remain conceptual or have been demonstrated only in non-ocular settings—a distinction that is often obscured in computational vaccinology literature. For most pathogen–technology combinations relevant to ocular vaccine development, the available evidence currently resides at levels 1–2, reflecting the early stage of the field and the substantial gap between *in silico* prediction and experimental or clinical validation.

**Table 3:** Evidence level framework and current status for key computational approaches in ocular vaccinology.

Pathogen	Computational approach	Evidence level	Key evidence basis	Critical gap
*C. trachomatis*	Pan-genome antigen discovery	L2	MOMP epitopes identified computationally and tested *in vitro*; partial animal model data (43, 85, 86)	No human immunogenicity or clinical ocular endpoint
*C. trachomatis*	ML epitope prediction (T-cell)	L1–L2	HLA-binding peptides predicted; limited *in vitro* validation (52, 86)	No ocular animal or human study
HSV-1	Structural vaccinology (gB/gD)	L3–L4	Preclinical herpetic keratitis models; some human Phase I systemic data (8, 20, 58, 59)	No demonstrated protection against recurrent keratitis
Adenovirus (EKC)	Computational serotype clustering; mosaic antigen design	L1–L2	*In silico* cross-serotype epitope mapping; genomic epidemiology of circulating serotypes (21, 68, 88)	No conjunctival sIgA data; no validated ocular animal model
*Acanthamoeba* spp.	Reverse vaccinology (surface proteome)	L1	Computational identification only; no experimental ocular validation (25, 67)	No correlates of protection defined in human disease
*O. volvulus*	Pan-genome; immunoinformatics	L1	*In silico* predictions; no validated ocular vaccine candidate (84)	No human challenge model; ethical and logistical constraints
Multi-pathogen	Digital twins (immune simulation)	L1	Conceptual framework; no ocular-specific validated application (107, 108)	No clinical or animal validation in ocular context
Multi-pathogen	AI delivery optimization	L1–L2	Computational nanoparticle modeling; *in vitro* ocular surface retention studies (70, 71)	No ocular animal or human clinical data
Multi-pathogen	Geospatial surveillance	L3–L4	Applied to trachoma mapping and EKC outbreak tracking (73–75)	Not yet linked to vaccine targeting or effectiveness outcomes

Evidence levels: L1, computational prediction only (*in silico*); L2, *in vitro*/biochemical validation; L3, ocular or relevant animal model data; L4, human immunogenicity data; L5, clinical protection against ocular outcomes.

Deep learning models, particularly convolutional and transformer-based architectures, have further advanced epitope prediction by integrating sequence context, structural features, and evolutionary information within a single framework (54). These models can be used to predict peptide–MHC binding, antigen processing, proteasomal cleavage, and epitope immunogenicity, thereby enabling a more comprehensive assessment of vaccine candidates (55). Machine learning–based epitope prediction can also support the design of multi-epitope and peptide vaccines tailored to HLA haplotype distributions or population-level allele frequencies, with the goal of improving coverage across diverse populations. However, these approaches remain limited by biases in training datasets, which are often enriched for well-characterized pathogens, HLA types, and study populations, underscoring the need for more diverse and representative immunological data (56).

Structural vaccinology is another area in which artificial intelligence is expanding the scope of rational vaccine design. Because antigen three-dimensional structure is central to antibody recognition and epitope accessibility, recent advances in computational structural biology, particularly AI-based protein structure prediction, have greatly increased access to high-quality structural models (57). These tools can support the identification of conformational epitopes, mapping of neutralizing antibody binding sites, and design of stabilized antigen conformations that preferentially present protective epitopes while reducing exposure of non-neutralizing or immunodominant but less protective regions. When combined with molecular dynamics simulations, AI-based structural models can also be used to examine antigen flexibility, stability, and epitope accessibility under physiologically relevant conditions (58). Such information is highly valuable for rational antigen engineering, including structural optimization, nanoparticle display, and fusion-protein design. Structural vaccinology has been especially influential in viral vaccine development, where structure-guided targeting of conserved or functionally constrained epitopes can help address antigenic variation. In this setting, AI can facilitate structure-guided antigen refinement before experimental validation, thereby reducing reliance on iterative trial-and-error optimization and accelerating translational development (59).

Beyond the analysis of individual antigens or epitopes, systems immunology provides a framework for understanding vaccine-induced immune responses as dynamic and interconnected networks. Vaccination triggers coordinated changes across multiple biological layers, including gene expression, protein abundance, cellular phenotypes, and metabolism. High-throughput omics technologies capture these responses at genomic, transcriptomic, proteomic, and metabolomic levels, generating multidimensional datasets that can be integrated through data science approaches to identify biologically meaningful patterns (60). Multi-omics analyses have been used to define molecular signatures associated with vaccine efficacy, durability, and adverse events (61). For example, early transcriptional responses in peripheral blood have been shown to predict subsequent antibody titers for several vaccines, suggesting the existence of conserved modules of vaccine-induced immunity. Machine learning can further enhance this approach by identifying latent features in high-dimensional datasets that may be difficult to detect using conventional statistical methods. Such predictive biomarkers may help optimize vaccine formulation, dosing, and adjuvant selection and may also support participant stratification in clinical trials (62).

Network-based methods further complement systems immunology by modeling interactions among genes, proteins, and immune cell populations, thereby capturing immune behavior as an emergent property of interconnected pathways rather than isolated molecular events. In vaccine research, such analyses can help identify network states and pathways associated with protective immunity as distinct from non-protective or pathological responses (63). This systems-level perspective may be particularly relevant in tissues with tightly regulated immune environments, where excessive inflammation can compromise tissue integrity. Despite these advantages, data science and AI approaches in vaccinology face important limitations (64). Model performance depends heavily on the quality, availability, annotation, and harmonization of underlying datasets, and computational predictions must be validated experimentally because biological complexity cannot be fully captured *in silico*. Additional challenges include interoperability across platforms and protocols, as well as ethical concerns related to data privacy, algorithmic bias, and equitable access to AI-enabled technologies (65). Nevertheless, ongoing advances in computational infrastructure, algorithm development, and collaborative data-sharing frameworks are helping to mitigate these barriers. Taken together, these developments support continued exploration of data-driven methods as tools to address key challenges in vaccinology, including improved vaccine design, delivery, and immune-response optimization.

### 4.2 Applications to ocular vaccine design, surveillance, and evaluation

The integration of data science, artificial intelligence, and immunology has created new opportunities to address longstanding challenges in vaccine development for ocular diseases (66). **Figure 3** outlines an end-to-end computational vaccinology pipeline, spanning pathogen and host data acquisition through rational vaccine design. Ocular infectious and inflammatory diseases have historically received limited attention in vaccinology, owing to the complexities of ocular immune regulation, the scarcity of high-quality clinical datasets, and the difficulty of extrapolating principles of systemic immunity to the ocular surface. In this context, data-driven approaches can support more rational antigen discovery, predictive modeling of ocular immune responses, and the design of delivery systems and adjuvants tailored to the distinctive ocular environment. A key opportunity lies in the *in silico* identification of novel antigens and conserved epitopes for ocular vaccine development (67). Genome-scale analyses of ocular pathogens can systematically prioritize proteins that are surface exposed, secreted, or otherwise accessible to host immune recognition. Comparative genomics and evolutionary modeling can further identify conserved regions maintained across strains despite immune pressure, making them attractive candidate vaccine targets with a lower predicted risk of immune escape (68). Computational epitope mapping can also refine candidate selection by predicting B-cell and T-cell epitopes with the greatest likelihood of immunogenicity across diverse human leukocyte antigen backgrounds (26). For highly variable ocular pathogens, including adenoviruses and *Chlamydia trachomatis*, these strategies may support the rational design of multivalent or mosaic antigens intended to maximize population coverage while minimizing redundancy (69).

**Figure 3:**
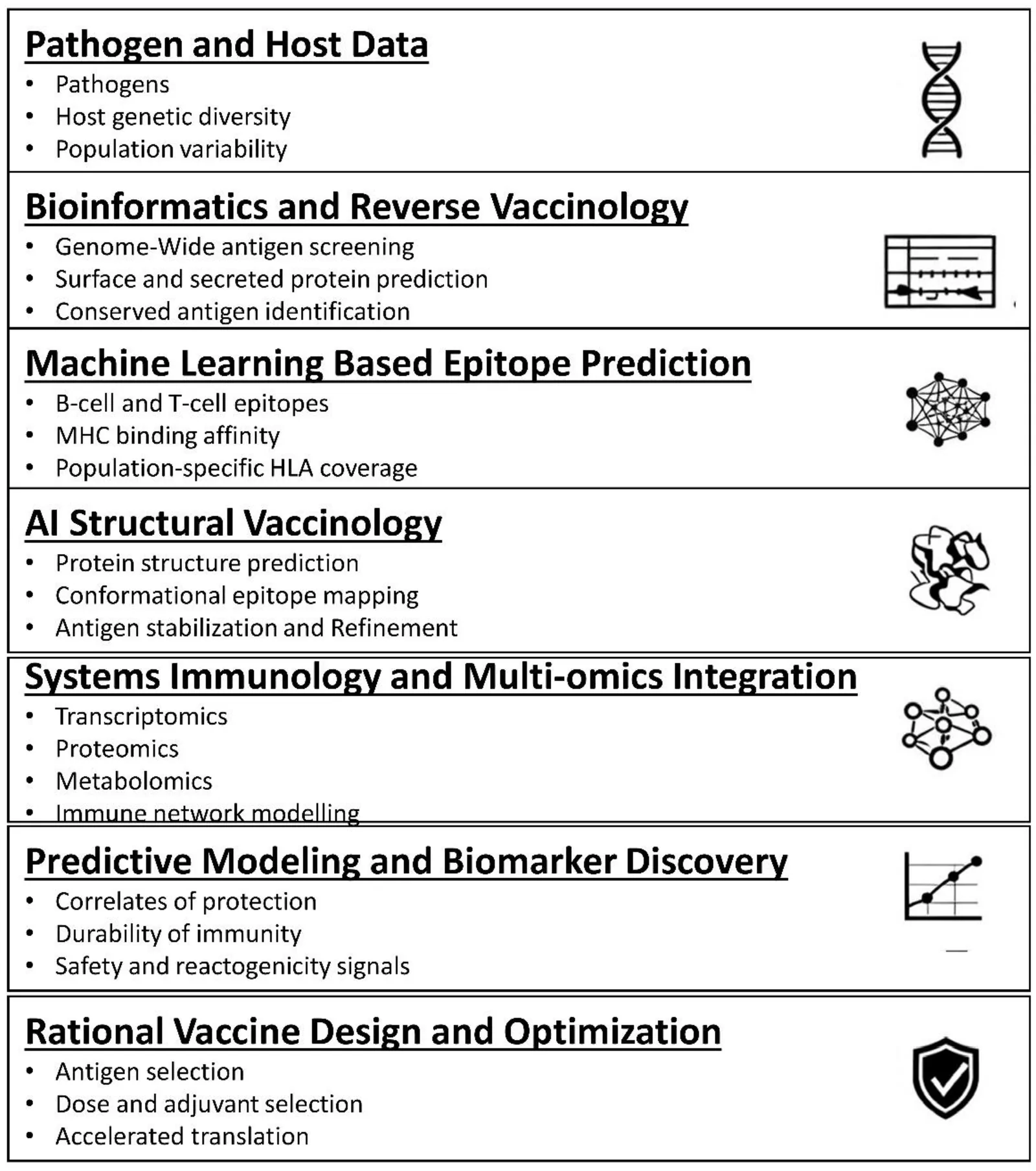
Integrated AI- and data-driven pipeline for modern vaccine development. The framework illustrates the progression from pathogen and host data through bioinformatics, machine learning–based epitope prediction, AI structural vaccinology, multi-omics integration, and predictive modeling, culminating in rational vaccine design and optimization.

Computational modeling is also expanding opportunities to design mucosal and targeted vaccine delivery systems better suited to the ocular environment. The ocular surface presents substantial physical and biological barriers, including tear turnover, epithelial tight junctions, and local immune-regulatory mechanisms, all of which can limit antigen retention and local immune induction (27). In this setting, simulations can be used to estimate how nanoparticle size, charge, composition, and surface functionalization influence retention time, tissue penetration, and antigen-release kinetics (70). Similar approaches may also inform the design of viral and non-viral delivery vectors by modeling parameters such as tissue tropism, duration of expression, and safety-related properties (71). More broadly, computational methods can support rational design of delivery platforms by integrating biophysical parameters with expected immunological outcomes, with the goal of enhancing local immunity while limiting systemic exposure and adverse effects. At the same time, predictions generated by AI-driven design pipelines require experimental and clinical validation before they can inform practice. Population-scale data, including surveillance, genomic, and clinical datasets, may then help translate these designs into actionable strategies for vaccine targeting, deployment, and long-term effectiveness assessment.

The expanding availability of large-scale epidemiological, clinical, and genomic datasets has substantially advanced infectious disease surveillance, interpretation, and public health decision-making. In ocular infectious disease research, big data approaches can provide insights that extend beyond those obtained from conventional surveillance systems and clinical trials (72). By integrating geospatial analytics, real-world clinical data, and genomic epidemiology, researchers and public health agencies can better identify disease hotspots, evaluate vaccine safety and effectiveness across heterogeneous populations, and monitor pathogen evolution that may compromise long-term protection (73). **Figure 4** presents a systems-level framework that links big data, genomic epidemiology, and real-world evidence to support evidence-based decision-making in ocular vaccinology. These approaches may be particularly valuable for ocular diseases, which often show geographic clustering, heterogeneous risk profiles, and incomplete capture in routine health systems.

**Figure 4:**
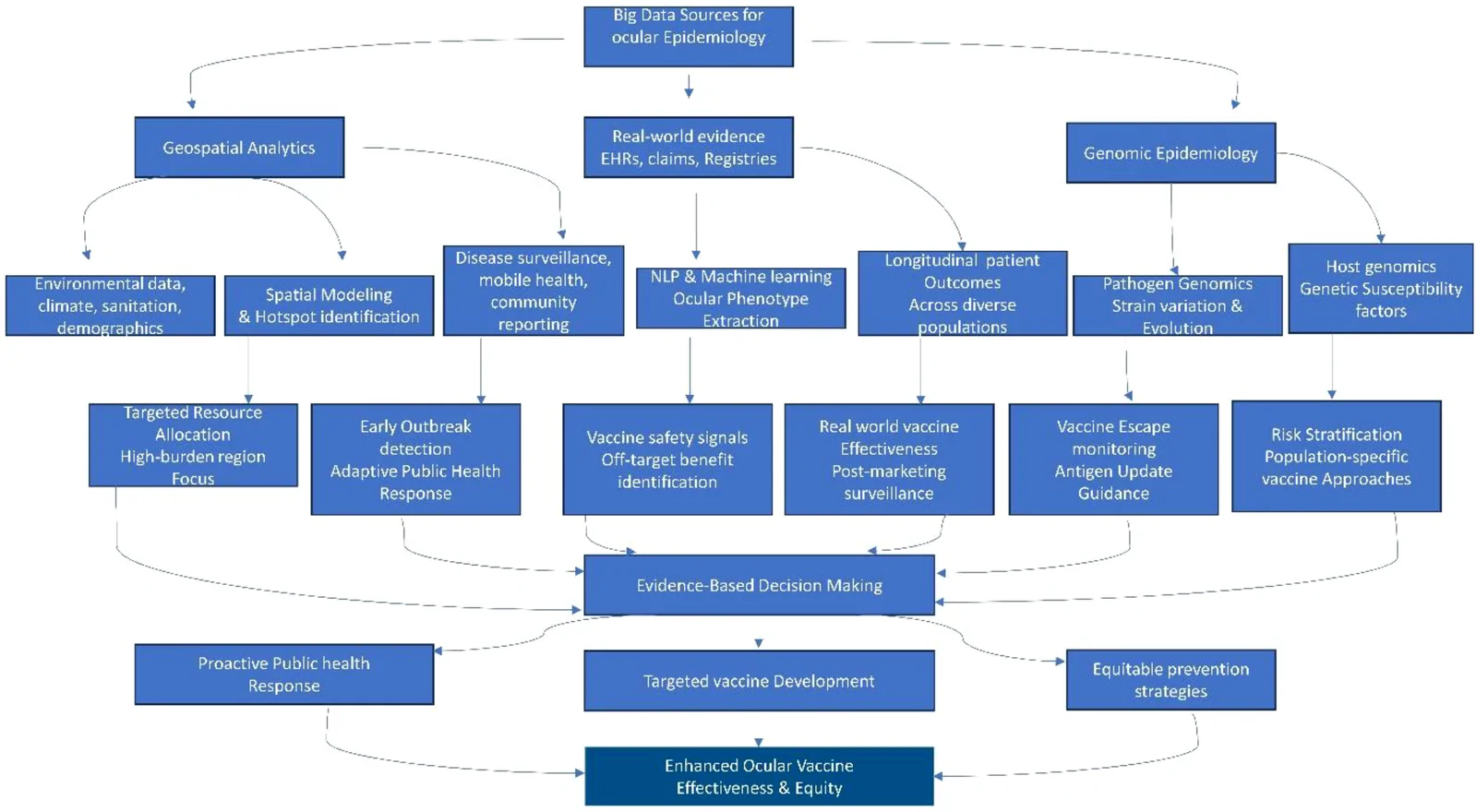
Big data–driven framework for ocular epidemiology and vaccine decision-making.

Geospatial analytics has become an important tool for mapping the distribution of ocular infections and identifying populations at increased risk. Environmental, socioeconomic, and behavioral determinants, including climate, sanitation, access to clean water, population density, and healthcare access, contribute substantially to the burden and distribution of diseases such as trachoma, onchocerciasis, fungal keratitis, and epidemic viral conjunctivitis (74). By integrating satellite imagery, climate variables, demographic information, and surveillance records, spatial models can detect fine-scale transmission patterns that are often obscured in aggregated national statistics. These insights can support more efficient resource allocation by prioritizing high-burden regions rather than relying exclusively on uniform population-wide strategies (75). In settings with constrained vaccine supply or major logistical barriers, geospatial prioritization may improve the impact and efficiency of targeted interventions.

In addition to spatial mapping, geospatial surveillance can be applied longitudinally and, in some settings, in near real time to monitor disease trends and evaluate the impact of interventions. Early warning of outbreaks of infectious ocular diseases, particularly viral conjunctivitis, may be supported by mobile health platforms, community-based reporting, and non-traditional data streams such as school absenteeism, internet search activity, or pharmacy sales (76). When integrated with vaccination coverage and other public health indicators, these systems may support adaptive response strategies that adjust to changing epidemiological conditions. Such approaches may be especially relevant for ocular diseases associated with high morbidity but relatively low mortality, which may be underdetected in conventional surveillance systems (77).

Another important opportunity for advancing ocular vaccine research lies in electronic health records and other sources of real-world evidence. Clinical trials for ocular vaccine applications are often constrained by ethical, logistical, and financial limitations, which can restrict sample size and duration. Data derived from electronic health records, insurance claims, and clinical registries can complement trial data by capturing outcomes in large and heterogeneous populations over longer periods (78). These datasets can support assessment of vaccine safety and real-world effectiveness, including rare adverse events, durability of protection, and outcomes in populations often underrepresented in trials, such as older adults, immunocompromised individuals, and patients with preexisting ocular disease (34).

Advanced data science approaches, including natural language processing and machine learning, can be used to extract clinically relevant ocular phenotypes from unstructured sources such as clinical notes, imaging reports, and diagnostic codes. This is particularly important in ophthalmology, where nuanced clinical descriptions and imaging findings are often incompletely captured by standardized coding systems (79). By linking vaccination histories with ophthalmic outcomes, these approaches may help identify protective associations, potential off-target effects, and ocular inflammatory safety signals. Such real-world data can also support post-marketing surveillance, regulatory evaluation, and iterative refinement of vaccination strategies as additional evidence accumulates (80).

Genomic epidemiology adds a complementary evolutionary dimension by enabling more detailed tracking of pathogen and host variation. Sequencing of ocular pathogens can characterize circulating strains, genetic diversity, and emerging variants with altered virulence or immune-evasion potential. For pathogens such as adenoviruses, herpesviruses, and *Chlamydia trachomatis*, genomic data can help define transmission networks, geographic distribution, and patterns of recombination and mutation that are relevant to vaccine design and long-term effectiveness. When integrated with epidemiological and clinical data, pathogen genomics may also support proactive surveillance for antigenic drift or potential vaccine escape and help guide timely updates to antigen composition (81).

Host genomic data can further strengthen epidemiological understanding by identifying genetic factors that influence susceptibility to ocular infection, disease severity, and vaccine responsiveness. Variation in immune-related genes, including HLA alleles and innate immune receptors, may contribute to interindividual and population-level differences in responses to both infection and vaccination (82). Incorporating host genetic information into epidemiological models may therefore improve risk stratification and support the development of population-tailored vaccine strategies, particularly in genetically diverse settings. At the same time, the use of host genomic data requires careful attention to privacy, consent, governance, and equitable implementation if it is to inform public health strategies responsibly (83). More broadly, the integration of geospatial analytics, real-world clinical data, and genomic epidemiology offers a framework for evidence-based decision-making in ocular vaccinology. Such complementary data streams may support a shift from reactive to more anticipatory public health responses by informing intervention strategies prospectively rather than relying solely on retrospective analysis. Even so, translation from predictive models to clinical impact is not automatic (84). The distinct biological, translational, and ethical challenges of ocular vaccinology, including immune privilege, the risk of inflammatory damage, and the difficulty of conducting trials in vulnerable populations, must be addressed systematically to ensure that computational advances lead to meaningful patient benefit.

## 5 Mechanistic determinants of computational vaccine translational success across ocular pathogen classes

A comparative mechanistic analysis of how computational approaches have performed across biologically distinct categories of ocular pathogens provides more generalizable and scientifically instructive conclusions than a sequential account of individual vaccine development programs. The four principal categories of primary ocular pathogens—obligate intracellular bacteria (*Chlamydia trachomatis*), latency-establishing DNA viruses (herpes simplex virus type 1), high-serotype-diversity non-enveloped DNA viruses (adenoviruses), and eukaryotic parasites with complex life cycles (*Acanthamoeba* species and *Onchocerca volvulus*)—each present a distinct combination of biological, immunological, and computational challenges that differentially determine the value and the limitations of data-driven vaccine design. Understanding these differences makes it possible to identify where computational approaches have generated actionable insights, where fundamental biological barriers remain intractable by current *in silico* methods, and what conditions must be met before predictions can be meaningfully translated toward experimental or clinical testing **Figure 5**. Critically, in each of these pathogen categories, the immune-privileged microenvironment of the eye introduces an additional layer of immunological complexity that is largely absent from the modeling assumptions underlying current computational vaccinology pipelines (**Table 4**) (25, 85).

**Figure 5:**
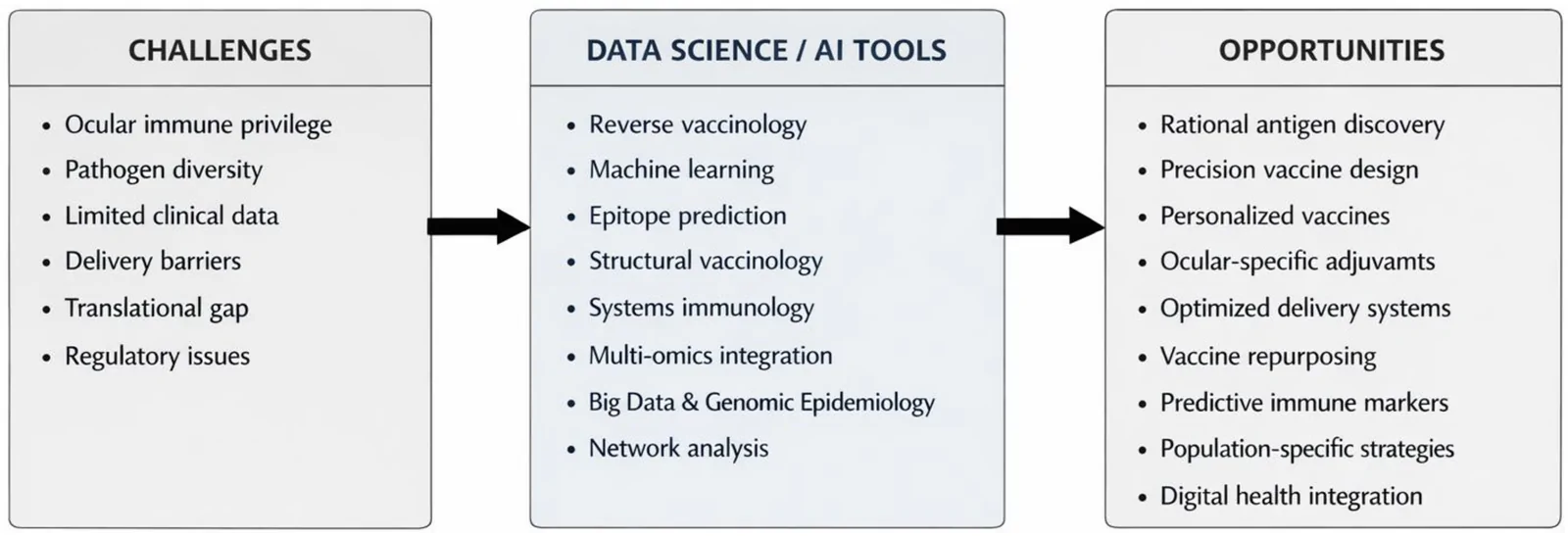
Conceptual framework integrating challenges, data science/AI tools, and opportunities in ocular vaccinology.

**Table 4:** Mechanistic comparison of computational vaccine design challenges, added value, and remaining gaps across major ocular pathogen classes.

Pathogen	Key pathogen biology	Immune mechanism required for protection	Where computational approaches add value	Remaining mechanistic gap
*Chlamydia trachomatis*	Obligate intracellular; biphasic EB/RB lifecycle; serovars A–C; MOMP surface-variable	CD4+ Th1 + local sIgA; Trm cells; avoidance of scarring-associated Th2/Th17	Pan-genome identifies conserved MOMP epitopes; ML predicts cross-reactive T-cell epitopes; transcriptomics distinguishes protective vs. pathological host responses	MOMP conformational epitopes resist linear prediction; protective-vs-pathological Th17 threshold not computationally defined; ACAID role at ocular surface uncharacterized
Herpes simplex virus type 1	dsDNA; trigeminal ganglion latency; periodic reactivation drives keratitis; tegument proteins immunodominant	CD8+ Trm cells in corneal epithelium and trigeminal ganglia; non-cytolytic antiviral function (IFN-γ, granzyme B)	Structural vaccinology maps conserved gB/gD neutralizing epitopes; transcriptomics of latency-associated transcripts identifies reactivation regulatory targets	Latency not reproducibly modeled *in silico*; Trm-mediated reactivation control correlates remain undefined
Adenoviruses (EKC)	Non-enveloped dsDNA; >30 serotypes associated with ocular disease; limited cross-neutralization; corneal stromal sequestration	Serotype-specific neutralizing sIgA; broad CD4+/CD8+ T-cell responses to conserved hexon	Computational serotype clustering enables mosaic antigen design; deep learning identifies cross-serotype hexon epitopes; genomic epidemiology maps circulating serotype distributions	Biophysical immunogenicity of mosaic antigens not predictable without experimental validation; conjunctival sIgA induction not optimized
*Acanthamoeba* spp.	Free-living amoeba; trophozoite/cyst dimorphism; contact lens-associated; cyst wall highly drug-resistant	Innate immune activation; anti-cyst antibody responses; T-cell effector mechanisms poorly defined	Reverse vaccinology identifies surface-exposed trophozoite antigens; structural modeling of cyst wall proteins defines candidate targets	Cyst proteome poorly annotated; no validated human correlates of protection; trophozoite-to-cyst transition not captured computationally

EKC, epidemic keratoconjunctivitis; EB, elementary body; RB, reticulate body; MOMP, major outer membrane protein; Trm, tissue-resident memory T cells; sIgA, secretory immunoglobulin A; ML, machine learning; ACAID, anterior chamber-associated immune deviation.

*Chlamydia trachomatis*, the causative agent of trachoma, exemplifies the class of ocular vaccine targets in which computational methods have been most productively applied, yet where fundamental mechanistic gaps continue to limit translational progress. Its obligate intracellular biphasic lifecycle, involving the infectious elementary body and the replicating reticulate body, complicates experimental antigen characterization and makes culture-independent genomic approaches particularly valuable (19). Pan-genome analyses of ocular *C. trachomatis* isolates have systematically defined both conserved and variable regions of the major outer membrane protein (MOMP) and polymorphic membrane proteins, informing rational antigen selection for cross-serovar coverage (42, 68, 86). Machine learning-based epitope prediction has been applied to prioritize candidate T-cell epitopes with broad HLA coverage and low predicted risk of cross-reactivity with host proteins, while systems-level transcriptomic analyses of host responses to chlamydial infection have been used to distinguish gene expression signatures associated with protective Th1 immunity from those associated with pathological conjunctival fibrosis (87). However, important mechanistic gaps remain. MOMP conformational epitopes critical to neutralizing antibody recognition are not adequately captured by linear sequence-based prediction. More importantly, the immunopathological threshold—the degree of Th17-mediated inflammation that drives scarring rather than protection—has not been computationally defined, and the role of anterior chamber-associated immune deviation in shaping responses to chlamydial antigens at the ocular surface remains uncharacterized. No licensed trachoma vaccine exists, and these mechanistic uncertainties define the highest-priority experimental questions for the field (42).

Herpes simplex virus type 1 presents a fundamentally different challenge: the primary target of protective immunity is not neutralization of circulating virions but maintenance of viral latency and suppression of reactivation in the trigeminal ganglion and corneal nerve terminals. This requires the sustained presence of non-cytolytic antiviral CD8+ tissue-resident memory T cells (Trm cells) expressing IFN-gamma and granzyme B within corneal epithelium and sensory ganglia, rather than high systemic antibody titers (20). Structural vaccinology has successfully identified conserved neutralizing epitopes on glycoproteins B and D as candidate targets for preventing primary infection, and transcriptomic analyses of latency-associated transcripts have begun to characterize the molecular programs regulating viral reactivation (58). However, latency itself—and the immunological equilibrium that keeps HSV-1 quiescent in the trigeminal ganglion—cannot currently be modeled *in silico*, and the correlates of Trm-mediated reactivation control remain experimentally undefined. The consequence is that computational pipelines can inform antigen selection for primary infection prevention but cannot yet address the recurrent keratitis that causes the majority of HSV-1-related corneal blindness. Adenoviral keratoconjunctivitis presents a contrasting computational challenge: the primary barrier is serotype diversity rather than immune evasion or latency. More than 30 adenoviral types have been associated with ocular disease, with limited cross-neutralizing immunity between serotypes (21). In this setting, computational serotype clustering and deep learning identification of cross-serotype conserved hexon epitopes have generated concrete, testable hypotheses for mosaic antigen design (68). Genomic epidemiology has provided clinically actionable insights into circulating serotype distributions across geographic settings, supporting rational antigen composition decisions for multivalent formulations (88). The remaining mechanistic gap is not analytical but experimental: the biophysical immunogenicity of computationally designed mosaic antigens cannot be predicted without validation in appropriate ocular immunization models, and the delivery characteristics needed to generate sufficient sIgA at the conjunctival surface remain unoptimized.

Eukaryotic parasitic pathogens, including *Acanthamoeba* species and *Onchocerca volvulus*, present the most computationally intractable vaccine challenges among major ocular pathogens, and they also illustrate the limits of *in silico* approaches most starkly. *Acanthamoeba keratitis* is associated with a trophozoite-to-cyst transition in which the resistant cyst stage, characterized by an extensively cross-linked cyst wall composed of cellulose and mannoproteins, renders the organism highly resistant to both antimicrobial treatment and immune clearance (24). Reverse vaccinology applied to *Acanthamoeba* genomic data has identified surface-exposed trophozoite proteins as candidate vaccine antigens, and structural modeling has been used to characterize cyst wall protein architecture. However, the *Acanthamoeba* cyst proteome remains poorly annotated, no validated correlates of protection have been established in human disease, and the immune mechanisms that would need to be engaged by a protective vaccine—whether innate activation, anti-cyst antibody responses, or T-cell-mediated killing—have not been experimentally defined with sufficient precision to calibrate computational predictions. For *O. volvulus*, where adult worms establish long-term persistence with substantial immunomodulatory capacity, the challenge is additionally complicated by the absence of appropriate *in vitro* culture systems and the ethical and logistical constraints on human challenge studies (85). These parasitic examples underscore a broader principle: computational tools can generate candidate hypotheses but cannot substitute for the foundational experimental immunology needed to define what a protective immune response looks like.

Taken together, this cross-pathogen mechanistic comparison reveals consistent determinants of where computational vaccinology adds translational value and where it remains insufficient. Computational approaches are most productive when pathogen antigenic diversity is high enough that exhaustive experimental screening is impractical, when sufficient host-response datasets are available to train models that distinguish protective from pathological immunity, and when structural constraints limit the epitope search space to a manageable and experimentally testable set of candidates. They lose predictive validity when protective immune correlates are undefined and cannot serve as model calibration targets, when the most immunologically critical epitopes are conformationally labile or dependent on posttranslational modifications not captured by sequence-based prediction, or when the immunological environment of the target tissue differs fundamentally from that used to train and validate the model. For every primary ocular pathogen discussed in this section, this last condition applies: the immune-privileged ocular microenvironment, including the functional consequences of ACAID, aqueous humor immunosuppression, and the blood-ocular barrier, is not incorporated into any current computational pipeline. Addressing this gap by developing ocular-specific immune simulation frameworks and collecting matched intraocular and peripheral immunological datasets from carefully designed clinical studies represents the most important translational research priority that emerges from this comparative analysis (25, 85, 89).

## 6 Translational barriers and ethical considerations

### 6.1 Translational attrition of computationally designed vaccine candidates: mechanistic basis and ocular-specific implications

Despite the rapidly growing literature reporting computationally designed multiepitope vaccine candidates, the overwhelming majority fail to progress beyond *in silico* or early *in vitro* stages, and very few have demonstrated clinical immunogenicity or protective efficacy. This translational attrition is now widely recognized as a systemic limitation of the field (57) and was specifically examined for *in silico*-designed multiepitope constructs in a 2026 analysis published in Frontiers in Immunology (90). Understanding the mechanistic basis of this failure is not peripheral: it is essential for calibrating the role of computational approaches in ocular vaccinology and for ensuring that their promise is not overstated relative to their current evidence base.

Several converging factors explain the gap between computational prediction and *in vivo* efficacy. First, epitope-prediction models carry well-documented dataset biases and capture MHC binding affinity but not the downstream attrition from proteasomal processing, central tolerance, and regulatory suppression—limitations discussed in detail in Section 4.1 (52, 55). Third, multiepitope vaccine constructs frequently suffer from steric interference between epitopes, linker-induced conformational artefacts, and unpredicted cross-reactivity with host proteins—none of which current *in silico* pipelines model adequately (12). Fourth, adjuvant selection, route of administration, and the local immunological microenvironment are critical determinants of vaccine outcome that are almost entirely absent from computational design frameworks: an optimized antigen may elicit markedly different—and potentially adverse—responses depending on delivery route and tissue context (53). Fifth, production feasibility, immunogen stability under field conditions, and regulatory manufacturability are rarely considered during computational candidate design, yet they are frequent barriers to late-stage development (57).

For ocular vaccinology specifically, these general translational barriers are compounded by two additional ocular-specific factors. First, the immune-privileged environment of the eye—including ACAID, aqueous humor immunosuppression, and the blood-ocular barrier—creates a local immune context that is not represented in any currently available computational model, as described in detail in Section 2. A vaccine candidate predicted to generate potent Th1 and antibody responses may instead trigger immune deviation or regulatory suppression when its antigens are encountered within the anterior chamber or ocular surface (31). Second, the immune correlates of protection remain undefined for most primary ocular pathogens: for trachoma, the precise balance of Th1 immunity, sIgA, and regulatory responses needed to prevent conjunctival scarring without driving immunopathology has not been established (91). Without empirically defined correlates of protection as calibration targets, computational tools cannot generate meaningful efficacy predictions for ocular applications. Together, these considerations underscore that *in silico* vaccine design should be treated as a hypothesis-generating and candidate-prioritization tool rather than a predictive pipeline for clinical efficacy. Computational predictions must be subjected to rigorous, phased experimental validation in immunologically appropriate ocular models—including ex vivo conjunctival or corneal tissue systems, ocular organoids, and appropriate *in vivo* models—before clinical translation can be meaningfully considered (92).

### 6.2 Data infrastructure, preclinical model limitations, and regulatory challenges in ocular vaccine translation

Developing computational approaches to support the design and evaluation of safe and effective vaccines for ocular infectious diseases remains challenging despite rapid advances in artificial intelligence, data science, and immunological modeling (51). Although many translational barriers are shared with AI-enabled biomedical research more broadly, ocular vaccinology presents additional difficulties related to the heterogeneity of ophthalmic data, the complexity of clinically defined endpoints, and the limited interoperability of imaging and electronic health record systems across centers (15). These barriers are especially relevant because ophthalmic research depends heavily on imaging modalities such as slit-lamp photography, optical coherence tomography, corneal topography, and fundus imaging, which are frequently stored in proprietary or non-uniform formats and graded with variable criteria (93). Cross-study integration is further complicated by inconsistent severity grading for conditions such as trachomatous scarring, microbial keratitis, and adenoviral keratitis infiltrates, as well as by the fact that many ocular infections are diagnosed and managed clinically without microbiological confirmation (94). Such heterogeneity can reduce model transportability, reproducibility, and, in some settings, predictive performance when algorithms are trained on non-harmonized datasets. Progress will therefore depend on standardized ophthalmic ontologies, interoperable imaging standards, shared repositories, and consensus-based clinical outcome definitions that enable robust computational-experimental translation (95).

Several dataset-specific limitations are particularly consequential for ocular vaccine research and warrant explicit discussion. First, training datasets for epitope-prediction models and immune simulation tools are heavily enriched for HLA supertypes prevalent in European-ancestry populations and include sparse representation of HLA diversity in sub-Saharan Africa, South Asia, and Southeast Asia—the regions carrying the highest burden of trachoma, onchocerciasis, and bacterial keratitis (52, 56). This creates systematic underperformance precisely in the populations where ocular vaccines are most urgently needed. Second, pathogens underrepresented in global genomic databases—including *Acanthamoeba* species, ocular tropic *Chlamydia trachomatis* serovars A–C, and emerging adenoviral types—generate proportionally less reliable computational predictions, because model accuracy scales with the volume and diversity of training data. Third, microbiological confirmation of ocular infections is inconsistent in clinical practice across settings: many cases of microbial keratitis and bacterial conjunctivitis are managed empirically without culture or molecular confirmation, and electronic health records frequently capture only clinical diagnoses without pathogen-level data (94). This limits the quality of real-world datasets available for training and validation of surveillance and effectiveness models. Fourth, the variation in acquisition protocols, device types, and grading criteria for ophthalmic imaging modalities—including slit-lamp photography, anterior segment OCT, and confocal microscopy—reduces cross-study comparability and the transportability of AI models trained on one imaging dataset to another (94). Fifth, and critically, external validation of computational models on geographically and demographically distinct populations remains the exception rather than the rule in ocular AI research (64). These limitations do not invalidate computational approaches but impose important constraints on their generalizability that must be communicated explicitly to avoid overestimation of their predictive accuracy.

A major translational challenge in ocular vaccinology is the need to model the ocular immune microenvironment accurately (25). The eye is an immune-privileged organ in which inflammatory responses are tightly regulated to preserve tissue integrity and visual function, particularly in vulnerable structures such as the cornea and retina (96). As a result, vaccine design for ocular pathogens must account not only for immunogenicity but also for the risk of inflammation-mediated tissue injury. This is especially important because immune correlates relevant at the ocular surface, including mucosal responses and tissue-resident memory T cells, may not be adequately captured by systemic immune models or by serum antibody measurements alone (89). Accordingly, more predictive frameworks will require ocular-specific immune modeling that incorporates local immune regulation, tissue-specific inflammatory thresholds, and spatially organized responses across ocular compartments (44). A related limitation is the imperfect availability of preclinical models that reproduce human ocular anatomy, tear-film biology, immune responses, and disease progression. Although mouse and other animal models have been informative for conditions such as herpes simplex keratitis and bacterial keratitis, important interspecies differences limit direct translation to humans (97). Similar limitations apply to trachoma and adenoviral keratoconjunctivitis, for which existing models capture only selected aspects of conjunctival scarring, transmission, or corneal sequelae rather than the full spectrum of human disease. Promising complementary approaches include human corneal organoids, ex vivo tissue systems, ocular organ-on-chip platforms, and better-characterized higher-order animal models that can improve mechanistic testing before clinical translation (98).

The regulatory implications of computational approaches in vaccine development require nuanced discussion. Computational antigen selection, epitope prediction, and immune modeling do not themselves create a separate regulatory category for the resulting vaccine products. Regulatory agencies evaluate vaccines based on established evidentiary requirements for safety, immunogenicity, and efficacy, irrespective of how candidates were initially identified. The regulatory challenges introduced by computational methods are therefore not categorical but procedural: they concern the reproducibility of computational pipelines, the transparency of algorithm design and training data, version control for models used across development stages, and the documented relationship between *in silico* predictions and the experimental evidence generated to test them (99, 100). Regulatory submissions involving computationally derived antigens or AI-assisted manufacturing processes will increasingly be expected to demonstrate that computational decisions were made with traceable logic, that predictions were validated with appropriate experimental and clinical evidence, and that model outputs are robust to changes in input data or algorithm version (87). For ocular vaccines specifically, these procedural requirements are compounded by the absence of validated clinical endpoints for many ocular infections, the lack of agreed correlates of protection, and the need for regulatory engagement at early development stages to define acceptable evidence pathways for novel local delivery routes. The most productive path forward for ocular vaccine developers using computational approaches is not to seek a separate regulatory framework but to engage proactively with regulators to define iterative validation standards that integrate computational and experimental evidence transparently.

## 7 Future directions

Future progress in ocular vaccinology will depend on translating conceptual advances into clearly defined and testable research priorities. Development of standardized, large-scale, multicenter datasets integrating ophthalmic imaging, microbiological data, and longitudinal clinical outcomes remains a foundational priority, as detailed in Section 6.2 (101, 102). A second research priority is the development of ocular-specific experimental and computational models that better capture the localized immune environment of the eye. Ocular-specific experimental systems—including corneal organoids, conjunctival explants, and eye-on-chip platforms described in Section 6.2—represent a near-term priority for validating computational predictions before *in vivo* testing (103). A more exploratory direction is the development of ophthalmic digital twins that integrate clinical, imaging, and immunological data to simulate patient-specific responses and guide hypothesis generation for vaccine formulation, dosing, or route of delivery in higher-risk groups (104). However, such applications remain early-stage and will require longitudinal multimodal datasets, rigorous biological calibration, and prospective clinical validation before routine use can be justified (105).

Another research priority is the rational design of mucosal- and ocular-targeted vaccine delivery systems capable of overcoming rapid tear clearance, improving antigen residence at the ocular surface, and eliciting protective local immunity without provoking harmful inflammation (106). Comparative studies should evaluate delivery platforms such as nanoparticles, hydrogels, and viral vectors, together with administration routes including topical, subconjunctival, and intranasal immunization, to determine which combinations provide the most favorable balance of retention, immunogenicity, and local tolerability (53). In parallel, population-stratified vaccine design may become increasingly relevant for genetically diverse, high-burden settings, particularly for pathogens with substantial antigenic variability such as *Chlamydia trachomatis* and adenoviruses, although the practical application of host-genetic stratification in ocular vaccinology remains an emerging goal rather than established practice (107). Digital surveillance platforms, tele-ophthalmology, and linked real-world data systems may further support outbreak detection, implementation monitoring, and post-vaccination safety and effectiveness assessment, especially in geographically dispersed populations (108). To translate these advances into clinical use, iterative validation frameworks will be required in which computational predictions are tested *in vitro*, evaluated in advanced ocular models, and then examined in clinical trials with disease-specific ocular endpoints under early and sustained regulatory engagement.

## 8 Conclusion

Ocular infectious diseases remain an important cause of preventable vision loss worldwide, yet vaccine development for eye-specific pathogens has lagged behind that for many systemic infections. Unlike prior descriptive surveys, this review uniquely constructs an evidence-based, challenge-driven framework that maps the immunological constraints of the ocular microenvironment to specific computational approaches and critically dissects both the promise and the translational attrition of *in silico* vaccine design strategies in an ocular context. This review identifies three central themes: the distinctive biology of the eye, including immune privilege and the risk of inflammation-mediated tissue injury, complicates vaccine design; emerging computational approaches, such as reverse vaccinology, machine learning, structural modeling, and systems immunology, can improve rational vaccine discovery; and major translational barriers, including fragmented ophthalmic datasets, limited experimental models, incompletely defined immune correlates of protection, and evolving regulatory requirements, continue to hinder clinical application. Progress in ocular vaccinology will therefore require a systems-level strategy that integrates standardized multicenter datasets, ocular-specific immune profiling, physiologically relevant experimental platforms, and iterative validation from computational prediction to clinical testing. Near-term priorities include harmonizing imaging and clinical outcome data, developing multimodal repositories with microbiological and longitudinal follow-up information, refining ocular-specific preclinical models, and evaluating vaccine candidates using clinically meaningful ocular endpoints. If addressed systematically, these advances could move ocular vaccinology toward a more predictive and evidence-based approach to preventing sight-threatening infections.
